# Gaseous Air Pollutants and Respirable Crystalline Silica Inside and Outside Homes at Brick Kilns in Bhaktapur, Kathmandu Valley, Nepal

**DOI:** 10.3390/ijerph191912431

**Published:** 2022-09-29

**Authors:** John D. Beard, Steven M. Thygerson, Alisandra Olivares, Jaxson E. Tadje, Selah Willis, James D. Johnston

**Affiliations:** Department of Public Health, Brigham Young University, Provo, UT 84602, USA

**Keywords:** brick kiln, brick worker, carbon dioxide, exposure assessment, household air pollution, international environmental health, international occupational health, Nepal, respirable crystalline silica

## Abstract

Household and ambient air pollution remain public health problems in much of the world. Brick kiln employees in Nepal may be particularly at risk of high air pollution exposures and resulting health effects due to high levels of outdoor air pollution, substandard housing, and indoor biomass cooking. We conducted a cross-sectional study of indoor and outdoor air pollution concentrations at workers’ homes at four fixed chimney Bull’s trench brick kilns in Bhaktapur, Kathmandu Valley, Nepal. We measured air concentrations of carbon monoxide (CO), carbon dioxide (CO_2_), nitrogen dioxide (NO_2_), sulfur dioxide (SO_2_), and respirable crystalline silica (SiO_2_; cristobalite, quartz, tridymite) using established methods and conducted a survey about characteristics of homes or samples that may be associated with air pollution concentrations. Geometric mean concentrations of CO, CO_2_, and SiO_2_ (quartz) were 0.84 ppm, 1447.34 ppm, and 6.22 µg/m^3^, respectively, whereas concentrations of all other air pollutants measured below lower detection limits. Most characteristics of homes or samples were not associated with air pollution concentrations. We found a positive association between the variable how long lived in house and SiO_2_ (quartz) concentrations, which may reflect sustained take-home exposure to SiO_2_ (quartz) over time. Interventions should focus on administrative controls to reduce take-home exposure to SiO_2_ (quartz) in this population.

## 1. Introduction

Brick workers in Nepal are a uniquely vulnerable population with regard to hazardous inhalation exposures. As a population, these workers experience almost constant exposure to hazardous air pollutants from (1) poor outdoor air quality in the Kathmandu Valley, (2) occupational brick dust containing respirable crystalline silica (SiO_2_), and (3) household air pollution (HAP) during non-working hours. Fine particulate matter (PM_2.5_) in the Kathmandu Valley regularly exceeds the 5.0 µg/m^3^ guideline recommended by the World Health Organization (WHO), often by an order of magnitude or more [1,2,3]. One prior study also documented occupational exposures to respirable crystalline SiO_2_ exceeding the permissible exposure limit (PEL) established by the U.S. Occupational Safety and Health Administration (OSHA) for all job categories that were studied [4]. Average exposures by job category ranged from 71–331 µg/m^3^. When brick workers are not working, they often live at the brick kiln in poorly constructed housing with limited ventilation [5]. Due to their impoverished conditions, brick workers and their families often use inexpensive solid fuels (e.g., wood) for cooking, which leads to high indoor HAP exposures [6,7]. The repeated daily triadic inhalation exposures likely explain why brick workers in Nepal report a high prevalence of symptoms consistent with a diagnosis of chronic obstructive pulmonary disease (COPD) [8].

Inhalation of HAP from burning solid fuels is a well-recognized risk factor for developing COPD, particularly among women, who spend more time with direct exposure to smoke from cooking fires [9,10]. Previous assessments of HAP in brick worker housing focused exclusively on PM_2.5_ and PM_2.5_ chemical constituents. Thygerson et al. sampled 16 homes at four different brick kilns in the Kathmandu Valley during daytime hours. The geometric mean (GM) indoor PM_2.5_ concentration did not differ significantly from the GM outdoor PM_2.5_ concentration, and both indoor and outdoor levels exceeded 180 µg/m^3^ [5]. A later study by Johnston et al. sampled PM_2.5_ over approximately 24 h. This study compared homes with wood cooking fires (*n* = 6) to those using cleaner-burning liquefied petroleum gas (LPG) cookstoves (*n* = 12) at a single brick kiln in Bhaktapur, Kathmandu Valley, Nepal [7]. Homes with wood cooking fires had significantly higher PM_2.5_ concentrations (GM: 541.14 µg/m^3^) than homes with LPG cookstoves (GM: 79.32 µg/m^3^) or outdoor air (GM 48.38 µg/m^3^). PM_2.5_ peaks in homes using wood fires occurred during morning and evening cooking times. Johnston et al., subsequently reported results of the chemical analyses of PM_2.5_ filters [6]. They identified several PM_2.5_ constituents, including aluminum, calcium, copper, iron, silicon, and titanium, above levels found to be associated with respiratory health effects in prior studies [11,12].

To date, we are not aware of any studies that have reported measures of other pollutants in brick workers’ homes, although the potential for those exposures exists. Likely exposures include carbon monoxide (CO), carbon dioxide (CO_2_), nitrogen dioxide (NO_2_), sulfur dioxide (SO_2_), and non-occupational respirable crystalline SiO_2_ dust. It is common among some brick kilns in the Kathmandu Valley to use “woods, recycled motor oils, coals, fuel oils, diesels, tires, trashes, and plastics for fuel” [13] (p. 185), which contributes to ambient levels of CO, CO_2_, NO_2_, and SO_2_ [13,14]. Brick worker housing is generally built using un-mortared brick walls with tin roofs, and relatively large openings between the bricks and other building materials. This loose construction allows easy infiltration of ambient air pollution into and out of homes [5]. Agro-residue burning and forest fires, automobile traffic, manufacturing (brick kilns), and cooking activities (kerosene, firewood, and LPG) are major sources of CO in the Kathmandu Valley [15,16,17]. Brick workers in Nepal live in homes with high occupant density, which may contribute to elevated levels of CO_2_ from exhaled breath [5]. NO_2_ is among the most common air pollutants and is primarily produced by man-made sources, including burning of fossil fuel [18,19]. SO_2_ results from the burning of coal and other fuels containing sulfur. SO_2_ can then dissolve in water vapor or interact with other gases to form acid, sulfates, or other respiratory particles [20]. SiO_2_ is the major component of most rocks and contains biologically active (respirable) particles measuring < 5 μm [21]. Considering the proximity of worker housing to their respective brick kilns, and the potential for dust to blow into brick worker housing through gaps in construction materials, the possibility exists for non-occupational, indoor exposure to SiO_2_ during non-working hours. Thus, our purpose in this study was to measure indoor CO, CO_2_, NO_2_, SO_2_, and respirable crystalline SiO_2_ (cristobalite, quartz, tridymite) in brick workers’ homes. Understanding these exposures may provide important knowledge related to respiratory and other diseases in this population of workers, and potential avenues to prevent future exposures.

## 2. Materials and Methods

### 2.1. Study Design

**Figure 1:**
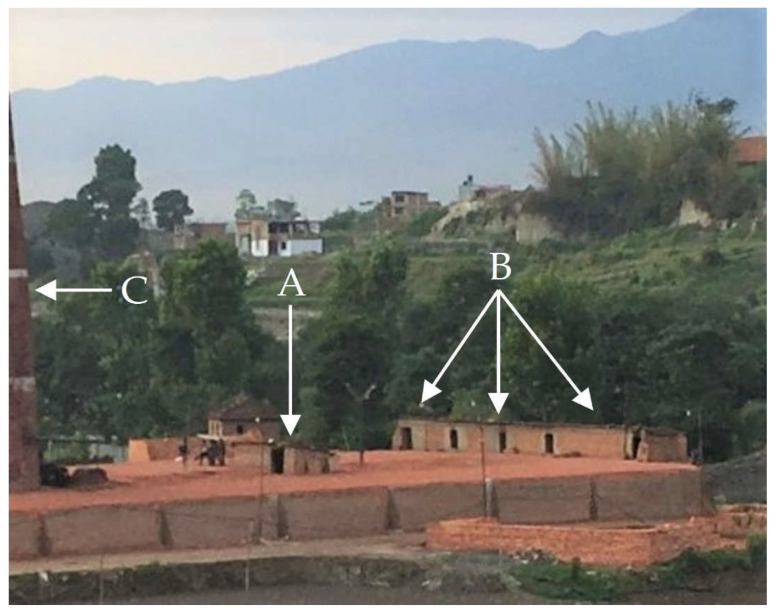
(A) Home of fire master on the brick kiln (for fixed chimney Bull’s trench brick kilns), (B) homes of brick workers adjacent to the brick kiln, and (C) brick kiln chimney in Bhaktapur, Kathmandu Valley, Nepal, May 2018.

We selected brick worker homes for our cross-sectional study using convenience sampling in May 2018. We recruited four homes from each of four brick kilns for a total of 16 homes. All homes included in our study were located within an approximate 100 m radius of their respective brick kiln, and all kilns were located in Bhaktapur, Kathmandu Valley, Nepal. At each of the four brick kilns, we characterized homes as being either fire master or worker domiciles. We then recruited two fire master and two worker homes at each kiln. Fire master homes are typically located on the brick kiln, while worker (non-fire master) homes are typically located adjacent to the kiln (Figure 1), which we hypothesized would lead to different exposure profiles. We hypothesized fire masters’ homes would have higher indoor and outdoor air pollution levels than workers’ homes due to their proximity to brick kiln smoke. The Institutional Review Board (IRB) at Brigham Young University reviewed this study prior to our arrival in Nepal. The IRB determined the study did not include human subjects’ research according to U.S. Code of Federal Regulations Title 45, Part 46 [22].

We collected samples indoors and outdoors at each study home. The arithmetic mean (AM) sampling time was 6.67 h (standard deviation [SD]: 0.38 h). We placed instruments by clipping them to a light-weight cord suspended between two tripod stands so that they hung about 1.1–1.2 m from the ground, the height of an adult home occupant’s mouth/nose when crouching inside the home, which was required due to the low heights of the roofs. The methods used for these samples, and a questionnaire we administered to an adult resident of each home regarding characteristics of homes or samples that may be related to air pollution concentrations, were described previously [5].

### 2.2. Respirable Crystalline SiO_2_ Measurement

Respirable crystalline SiO_2_ samples were collected in accordance with U.S. National Institute for Occupational Safety and Health (NIOSH) Method 7500 [23]. All SiO_2_ samples were collected using SKC AirLite (SKC Inc., Eighty Four, PA, USA) sampling pumps. Pumps were calibrated to 2.5 L/min prior to being deployed. Pumps were pre- and post-calibrated with a DryCal^®^ Defender 510 volumetric flow standard (Mesa Labs, Butler, NJ, USA). The sampling train consisted of tubing, a 37 mm styrene cassette housing a pre-weighed 5.0 μm PVC membrane filter, and an aluminum cyclone with a 4.0-μm cut-point (SKC Inc., Eighty Four, PA, USA). Pre- and post-air flow calibrations were conducted with the sampling train in-place. Gravimetric and X-ray diffraction (XRD) analyses for SiO_2_ were conducted at ALS Laboratories in Salt Lake City, UT, USA. The lower detection limit masses for cristobalite, quartz, and tridymite were five µg, five µg, and 30 µg, respectively.

### 2.3. CO and CO_2_ Measurement

We measured CO and CO_2_ levels using Drager Pac 7000 Personal Gas Detectors (SKC Inc., Eighty Four, PA, USA). Both the CO and CO_2_ monitors were pre-calibrated by SKC Inc. prior to sample collection (CO LDL concentration: 6 ppm; CO_2_ LDL concentration: 3000 ppm). Prior to each sampling day, we used the Pac 7000 software to ensure that the settings were correct and that the memory was clear on each device. We clipped a CO and CO_2_ device on the suspension string and followed the device manual for use (SKC Inc., Eighty Four, PA, USA). We set each device to measure in 10 s intervals. We had one additional CO and CO_2_ monitor saved for backup as needed. For post sampling, we connected each device to a computer through the Pac 7000 cartridge and then transferred and saved the data as Excel spreadsheets.

### 2.4. NO_2_ and SO_2_ Measurement

We assessed NO_2_ and SO_2_ levels using UME^X^200 Passive Samplers (SKC Inc., Eighty Four, PA, USA). Each badge is fitted internally with a tape treated with triethanolamine (TEA) that absorbs nitrogen oxides (NO_x_) and sulfur oxides (SO_x_). Following the instructions from SKC Inc., we opened the aluminum pouch to remove the badge, clipped it onto the suspension string, and slid the sampler cover down to the “on” position. After the sampling period, we slid the cover up to the “off” position and immediately enclosed the sampler in the original aluminum pouch (SKC Inc., Eighty Four, PA, USA). We took a field blank for each day of sampling. We stored the samples in the aluminum pouches until we sent them for analysis at ALS Laboratories in Salt Lake City, Utah, USA. The LDL masses were 2.5 and 1.8 μg for NO_2_ and SO_2_, respectively.

### 2.5. Statistical Analyses

We used SAS version 9.4 (SAS Institute, Inc., Cary, NC, USA) for all data management and analyses. CO and CO_2_ measurements were recorded every 10 s, so we calculated the AM of measurements during the sampling period for each sample. If 100% of CO or CO_2_ measurements were above the LDL for a particular sample, then we calculated the AM for the sample using intercept only linear regression models. If less than 100% of CO or CO_2_ measurements were above the LDL for a particular sample, then we calculated the AM for the sample using intercept only Tobit regression models. If the AM for the sample was below the LDL, then we considered the entire sample to be below the LDL.

For categorical characteristics of homes or samples, we calculated frequencies and percentages. For continuous characteristics of homes or samples, we calculated the AM, SD, minimum, first quartile, median, third quartile, and maximum. For air pollutants, we calculated frequencies and percentages of samples that had measurements above and below LDLs. For air pollutants that had at least one measurement above LDLs, we calculated GMs and 95% confidence intervals (CI) for each air pollutant using separate intercept only Tobit regression models that used the natural logarithm of air pollution concentrations as dependent variables because no air pollutant had 100% of measurements above LDLs and distributions of air pollutants were right skewed. We calculated minimum and maximum air pollutant concentrations from samples that had measurements above LDLs.

We used separate simple (i.e., unadjusted) exact unconditional logistic regression models to calculate exact odds ratios (OR) and exact 95% CI for associations between characteristics of homes or samples and having air pollutant concentrations above LDLs if greater than 10%, but less than or equal to 30%, of samples had measurements above LDLs [24,25]. We used separate simple (i.e., unadjusted) Tobit regression models that used the natural logarithm of air pollution concentrations as dependent variables to calculate GM and 95% CI for associations between characteristics of homes or samples and air pollutant concentrations if greater than 30% of samples had measurements above LDLs [24,25]. If a characteristic of homes or samples that had more than two categories was significantly associated with air pollutant concentrations, then we used the Tukey–Kramer method to test for pairwise differences in air pollutant concentrations among the categories of the characteristic of homes or samples.

We repeated analyses of associations between characteristics of homes or samples and air pollutant concentrations or having air pollutant concentrations above LDLs using indoor air samples only. We used multivariable Tobit regression models that used the natural logarithm of air pollutant concentrations as dependent variables to estimate adjusted associations between characteristics of homes or samples and air pollutant concentrations when more than one characteristic of homes or samples was associated with air pollutant concentrations in simple (i.e., unadjusted) Tobit regression models.

## 3. Results

We collected 32 air pollution samples from 16 homes (i.e., from four homes each at four kilns (Table 1). We collected 16 of the samples at homes of fire masters and 16 at homes of workers. Similarly, we collected 16 of the samples inside homes and 16 outside homes. The AM size of house was 82.16 ft^2^ (7.64 m^2^), the median time lived in house was 5.50 months, the AM number of people who lived in house was 4.71, and the median occupant density was 62.50 residents/100 m^2^. We collected 36% of samples at homes that had 1–3 children 0–18 years-old who lived in the house and 31% of samples at homes that had 1–3 children under six years-old who lived in the house. We collected 60%, 67%, and 50% of samples at homes that used wood only as the primary fuel for cooking, electricity as the type of heating source in the home, and candle as the type of non-electric light source in the home, respectively. We collected 67% of samples at homes that had any smokers living in the home and 33% of these samples at homes that had 2–4 smokers living in the home who regularly smoked inside the home. We collected 25% of samples at homes that had 3–4 smokers living in the home.

All samples had air concentrations below LDLs for NO_2_, SiO_2_ (cristobalite), SiO_2_ (tridymite), and SO_2_ (Table 2). Only two (6%), eight (25%), and 19 (61%) samples had concentrations of CO, CO_2_, and SiO_2_ (quartz), respectively, above LDLs. GMs were 0.84 ppm, 1447.34 ppm, and 6.22 µg/m^3^ for CO, CO_2_, and SiO_2_ (quartz), respectively.

Using all indoor and outdoor air samples, no characteristic of homes or samples was significantly associated with having CO_2_ concentrations above the LDL (Table 3). A two month increase in how long lived in house was significantly (*p* = 0.01) associated with a nine percent (95% CI: 2%, 18%) increase in GM SiO_2_ (quartz) concentrations (Table 4). Samples at homes with other (lightbulb, line cable) or no type of heating source in the home had significantly (*p* = 0.0008) higher SiO_2_ (quartz) concentrations (GM: 10.15, 95% CI: 6.84, 15.07 µg/m^3^) than samples at homes with electricity (GM: 3.77, 95% CI: 2.48, 5.75 µg/m^3^). Samples at homes with no smokers living in the home had significantly (*p* = 0.03) higher SiO_2_ (quartz) concentrations (GM: 9.36, 95% CI: 5.78, 15.13 µg/m^3^) than samples at homes with smokers (GM: 4.75, 95% CI: 3.16, 7.14 µg/m^3^). How many smokers living in the home was significantly (*p* = 0.03) associated with SiO_2_ (quartz) concentrations. Tukey–Kramer pairwise comparisons indicated samples at homes with zero smokers living in the home had significantly (*p* = 0.01) higher SiO_2_ (quartz) concentrations (GM: 9.43, 95% CI: 6.04, 14.75 µg/m^3^) than samples at homes with 1–2 smokers (GM: 3.53, 95% CI: 1.91, 6.54 µg/m^3^). No other characteristic of homes or samples was significantly associated with SiO_2_ (quartz) concentrations.

Using indoor air samples only, the exact OR for the association between a two month increase in how long lived in house and having CO_2_ concentrations above the LDL was 0.05 (exact 95% CI: < 0.01, 1.00; exact *p* = 0.05; Supplementary Materials, Table S1). Samples at homes with other (lightbulb, line cable) or no type of heating source in the home had significantly (*p* = 0.04) higher SiO_2_ (quartz) concentrations (GM: 9.74, 95% CI: 5.70, 16.66 µg/m^3^) than samples at homes with electricity (GM: 4.64, 95% CI: 2.86, 7.53 µg/m^3^; Supplementary Materials, Table S2). No other characteristic of homes or samples was significantly associated with having CO_2_ concentrations above the LDL or SiO_2_ (quartz) concentrations indoors. However, the aforementioned associations between characteristics of homes or samples and SiO_2_ (quartz) concentrations using all indoor and outdoor air samples were generally in the same directions and of similar magnitudes using indoor air samples only.

When we mutually adjusted how long lived in house, type of heating source in the home, and any smokers living in the home for each other using all indoor and outdoor air samples, only type of heating source in the home remained significantly (*p* = 0.0002) associated with SiO_2_ (quartz) concentrations. Similarly, only type of heating source in the home remained significantly (*p* = 0.002) associated with SiO_2_ (quartz) concentrations when we mutually adjusted how long lived in house, type of heating source in the home, and how many smokers living in the home for each other using all indoor and outdoor air samples.

## 4. Discussion

In our study of gaseous air pollutants and respirable crystalline SiO_2_ concentrations inside and outside homes of workers at brick kilns in Bhaktapur, Kathmandu Valley, Nepal, we found concentrations of air pollutants to be low and usually undetectable. Most characteristics of homes or samples were not associated with CO_2_ or SiO_2_ (quartz) concentrations using all indoor and outdoor samples or indoor samples only. Type of heating source in the home was the characteristic of homes or samples that was most consistently associated with SiO_2_ (quartz) concentrations in various analyses and concentrations were higher at homes with other (lightbulb, line cable) or no type of heating source in the home compared to homes with electricity. An explanation for why other or no type of heating source was associated with respirable SiO_2_ in brick workers’ homes is elusive. One thought is that the respirable fraction of SiO_2_ has a long settling time. It is possible that brick workers with no heating source behave differently during cold morning hours than workers with a heating source. For instance, workers with no heating may use more blankets that emit dust particles, resuspending small SiO_2_ particles in the air. For particles < 2.5 µm, it takes 85 min to fall one m, and for particles < 1.0 µm, it takes 485 min to fall one m in indoor air with low air exchange rates [26]. It may be that workers with no heating suspend more dust before leaving their homes for work, which we picked up on the air monitors.

We are unaware of previous studies that measured concentrations of gaseous air pollutants and respirable crystalline SiO_2_ inside or outside homes of brick kiln workers. However, a study of occupational exposures conducted at 12 brick kilns in Patiala district, Punjab, India, reported mean area air concentrations of 2.02 µg/m^3^ for CO, 0.89 µg/m^3^ for CO_2_, 96 µg/m^3^ for NO_x_, and 91.76 µg/m^3^ for SO_x_ [27]. Pangtey et al. conducted a study at 22 fixed chimney Bull’s trench brick kilns in Lucknow, India, and found mean area air concentrations of 3.44 ppm for CO, 0.23 ppm for NO_x_, and 0.35 ppm for SO_2_ [28]. A study conducted at three fixed chimney Bull’s trench brick kilns in Kasur district, Pakistan, used area samples to measure air pollution concentrations in two areas of the kiln: modulation and kiln [29]. Workers in the modulation area completed activities such as digging, wetting, mixing, and lifting clay and mud and molding and arranging bricks for drying, whereas workers in the kiln area completed activities such as carrying, loading, and arranging bricks, adding coal to the fire, and unloading and sorting fired bricks. Mean concentrations of NO_2_ and SO_2_ were 0.0591 ppm and zero ppm, respectively, in the modulation area and 0.07 ppm and 0.0652 ppm, respectively, in the kiln area. Sanjel et al. used personal breathing zone sampling to measure respirable crystalline SiO_2_ (quartz) among 46 workers at fixed chimney Bull’s trench brick kilns in the Kathmandu Valley, Nepal. The GM respirable crystalline SiO_2_ (quartz) concentrations among workers in five similar exposure groups were 92 µg/m^3^ for coal crushing/carrying preparation, 102 µg/m^3^ for fireman, 71 µg/m^3^ for green brick molding, 223 µg/m^3^ for green brick stacking/carrying, and 331 µg/m^3^ for red brick loading/carrying [4]. In our study, GM area air concentrations inside and outside homes of workers at four fixed chimney Bull’s trench brick kilns in Bhaktapur, Kathmandu Valley, Nepal, were 0.84 ppm for CO, 1447.34 ppm for CO_2_, and 6.22 µg/m^3^ for SiO_2_ (quartz), but NO_2_, SO_2_, SiO_2_ (cristobalite), and SiO_2_ (tridymite) concentrations were below LDLs. Considered together, these studies suggest air concentrations of CO, CO_2_, NO_2/x_, and SO_2/x_ at fixed chimney Bull’s trench brick kilns and on-site worker housing in Asia are typically low, whereas concentrations of SiO_2_ (quartz) are typically high at brick kilns, but low at on-site worker housing.

CO_2_ is an essential byproduct of cellular metabolism with ambient levels generally about 380 ppm [30,31]. Combustion of fossil fuels such as coal, natural gas and oil are the primary human activities that emit CO_2_. The main sources of CO_2_ emissions in the United States include gasoline and diesel emissions, electricity, and fossil fuel combustion during various industrial processes [32]. In Nepal, fossil fuel and biofuel consumption are the main sources of CO_2_ emissions and brick kilns generate an estimated 11% of CO_2_ emissions [33]. In our study, individuals mainly used wood for cooking, electricity as a heating source in the home and candles as a type of non-electric light source. The latest OSHA standard for CO_2_ is 5000 ppm as an eight-hour time-weighted average (TWA) concentration [34]. Concentrations as low as 800 ppm can increase heart rate, blood pressure, and cardiac output and stimulate ventilation and other respiratory symptoms while concentrations from 1000–2500 ppm can lead to significantly reduced cognitive performance [30,31]. Unconsciousness or death can result from exposure to concentrations of 10% (100,000 ppm) or more [34]. There are multiple ways to prevent harmful CO_2_ exposure including alarms which monitor CO_2_ concentrations in the home. In addition, educational interventions can help inform individuals of the hazards of CO_2_ in the air and prevent dangerous situations.

The SiO_2_ exposure experienced by our study population is directly caused by the bricks in Nepal’s brick kilns [4]. Inhalation exposure to crystalline SiO_2_ is associated with significantly increased risks of developing acute and chronic silicosis, COPD, pulmonary tuberculosis, and rheumatoid arthritis [35]. Among these, the health effect of highest concern is silicosis because the effects of SiO_2_ on the lungs are irreversible and can lead to lung cancer [36]. There is evidence that workers’ cumulative SiO_2_ exposure is directly associated with lung cancer risk [37]. The World Health Organization’s International Agency for Research on Cancer has declared crystalline SiO_2_ to be a group 1 (definite) human lung carcinogen based its review of both animal and human SiO_2_ exposure studies [38]. A no-observed-adverse-effect level (NOAEL) for SiO_2_ has not been established due to workers developing silicosis even at the lowest estimated cumulative exposure ranges reported [36]. To protect against these adverse health outcomes, OSHA has developed a PEL of 50 µg/m^3^ over an eight-hour work period and an action level of 25 µg/m^3^ [39]. If measured SiO_2_ levels rise above the action level, then the use of wet methods (e.g., wet spray misting) have been shown to be low-cost, effective methods to decrease exposure [40]. If the use of wet methods is not practical or feasible, then it is recommended that particulate respirators with a high-efficiency filter be worn by those experiencing exposure [41].

CO_2_ and SiO_2_ (quartz) concentrations were not associated with most characteristics of homes or samples in our study. Notably, location of sample (indoor, outdoor) was not associated with having CO_2_ concentrations above the LDL and indoor GM SiO_2_ (quartz) concentrations were not significantly different from outdoor GM SiO_2_ (quartz) concentrations. As discussed previously [5], the homes of brick kiln workers we sampled for our study were made of un-mortared bricks that had gaps between the bricks and the walls and tin roofs. The doors were typically covered by natural fiber hangings and the floors were made of dirt. This construction likely resulted in free exchange of air and air pollution between the inside and outside of homes and suspension and resuspension of dust into the breathing zones of brick workers and their family members. In addition, dust containing SiO_2_ (quartz) could have blown into brick workers’ homes and led to non-occupational, indoor exposure to SiO_2_ (quartz) during non-working hours.

Only a few characteristics of homes or samples were associated with SiO_2_ (quartz) concentrations in our study. A possible explanation for the positive association between how long lived in house and SiO_2_ (quartz) concentrations may be take-home exposure (e.g., via contaminated worker clothing). We detected SiO_2_ (quartz) in 61% of air samples collected at homes of brick kiln workers. Although the low GM SiO_2_ (quartz) concentration we found may suggest take-home exposure to SiO_2_ (quartz) is a relatively minor problem in this population of workers and their families, take-home exposures could result in SiO_2_ (quartz) dust accumulating at the home over time. As mentioned previously, the SiO_2_ (quartz) could then be resuspended into the breathing zones of brick kiln workers and their family members, which could be confirmed by future studies. Reasons for associations between type of heating source in the home and whether and how many smokers were living in the home and SiO_2_ (quartz) concentrations are not apparent. However, only type of heating source in the home remained significantly associated with SiO_2_ (quartz) concentrations when we restricted to indoor samples only or when we mutually adjusted associations between these characteristics and SiO_2_ (quartz) concentrations for each other.

Our study had several strengths and limitations. Strengths included a novel research question because we think we were the first to study concentrations of gaseous air pollutants and respirable crystalline SiO_2_ at homes of brick kiln workers. We also collected air samples at homes of brick workers at four different brick kilns in different areas of Bhaktapur, Kathmandu Valley, Nepal. We used state-of-the-art air sampling equipment, measured multiple air pollutants inside and outside of homes, and collected information about several characteristics of homes or samples which may be associated with air pollution concentrations. Limitations included our use of a cross-sectional study design with sampling at one point in time, so our results may not generalize to air pollution concentrations over longer periods of time. In addition, we used a convenience sample, so our results may not generalize to homes of other brick workers at the brick kilns included in our study or elsewhere. We had a relatively small sample size (*n* = 32 air samples), although it was 60% larger than other studies of air pollution conducted among this population [6,7]. We also sampled during non-prime cooking hours, which may explain why we found concentrations of air pollutants to be low and usually undetectable. In addition, the LDL for CO_2_ was relatively high. Finally, we used area rather than personal air samples, so our estimates of air pollution concentrations may not reflect those in study participants’ personal breathing zones. Future studies should address these limitations by measuring air pollution concentrations over longer periods of time (e.g., days to weeks or even months), including a larger, representative sample of homes or workers, sampling during prime cooking hours during the morning and evening, sampling during the colder season, using instruments that can measure air pollution, particularly gaseous pollutants, at lower concentrations, and using personal breathing zone sampling to estimate personal rather than area exposures.

## 5. Conclusions

In conclusion, concentrations of gaseous air pollutants and respirable crystalline SiO_2_ inside and outside homes of workers at brick kilns in Bhaktapur, Kathmandu Valley, Nepal, were low and usually undetectable. However, these results need to be confirmed by future studies that sample during prime cooking hours. Concentrations of CO_2_ and SiO_2_ (quartz) were not associated with the majority of characteristics of homes or samples. Take-home exposures to SiO_2_ (quartz) could lead to higher SiO_2_ (quartz) air concentrations at brick kiln workers’ homes over time. Interventions should focus on administrative controls such as providing brick kiln workers with clean clothing to use for non-work times and encouraging regular bathing, changing from work to non-work clothes at the end of work shifts, washing of work clothes, and cleaning of homes.

## Figures and Tables

**Table 1:** Characteristics of air pollution samples collected inside and outside homes at brick kilns in Bhaktapur, Kathmandu Valley, Nepal, May 2018.

Characteristic	Samples, *n*	Missing, *n*	AM	SD	Min	Q1	Median	Q3	Max
Total	32								
Size of house, feet^2^	28	4	82.16	36.39	20.00	52.00	84.00	112.00	150.00
Size of house, m^2^	28	4	7.64	3.35	1.90	4.80	7.80	10.40	13.90
How long lived in house, months	28	4	8.82	7.89	4.00	5.00	5.50	7.00	30.00
How many people live in house	24	8	4.71	2.19	2.00	3.00	4.00	6.00	9.00
Occupant density, residents/100 m^2^	22	10	73.48	49.89	19.23	43.17	62.50	90.91	210.53

Abbreviations: AM, arithmetic mean; Max, maximum; Min, minimum; Q1, first quartile; Q3, third quartile; SD, standard deviation.

**Table 2:** LDLs and summary statistics for air pollutants measured inside and outside homes at brick kilns in Bhaktapur, Kathmandu Valley, Nepal, May 2018.

	Total Samples								
					Above LDL				
Air Pollutant	LDL Mass (μg)	LDL Concentration Range	Missing, *n*	Below LDL, *n* (%)	*n* (%)	GM ^a^	95% CI ^a^	Min ^b^	Max ^b^
CO, ppm	NA	6.00		30 (94)	2 (6)	0.84	0.05, 14.00	9.91	12.99
CO_2_, ppm	NA	3000.00		24 (75)	8 (25)	1447.34	651.18, 3216.95	3799.32	12,975.99
NO_2_, μg/m^3^	2.50	325.47, 404.79		32 (100)	0 (0)	NA	NA	NA	NA
SiO_2_ (cristobalite), μg/m^3^	5.00	4.55, 6.04	1	31 (100)	0 (0)	NA	NA	NA	NA
SiO_2_ (quartz), μg/m^3^	5.00	4.55, 6.04	1	12 (39)	19 (61)	6.22	4.56, 8.48	5.19	43.28
SiO_2_ (tridymite), μg/m^3^	30.00	27.29, 36.22	1	31 (100)	0 (0)	NA	NA	NA	NA
SO_2_, μg/m^3^	1.80	266.71, 331.71		32 (100)	0 (0)	NA	NA	NA	NA

Abbreviations: CO, carbon monoxide; CO_2_, carbon dioxide; CI, confidence interval; GM, geometric mean; LDL, lower detection limit; Max, maximum; Min, minimum; NA, not applicable; NO_2_, nitrogen dioxide; SiO_2_, crystalline silica; SO_2_, sulfur dioxide. ^a^ Estimated using intercept only Tobit regression models of the natural logarithm transformed values. ^b^ Calculated using only samples that had values above LDLs.

**Table 3:** Associations between characteristics and having CO_2_ air concentrations measured inside and outside homes above the LDL at brick kilns in Bhaktapur, Kathmandu Valley, Nepal, May 2018.

	CO_2_, ppm				
Characteristic	Below LDL, *n* (%)	Above LDL, *n* (%)	Exact OR ^a^	Exact 95% CI ^a^	Exact *p*-Value ^a^
Kiln number					
1	6 (25)	2 (25)	1.00	Reference	
2	6 (25)	2 (25)	1.00	0.05, 18.27	
3	6 (25)	2 (25)	1.00	0.05, 18.27	
4	6 (25)	2 (25)	1.00	0.05, 18.27	1.00
Type of home					
Worker	13 (54)	3 (38)	1.00	Reference	
Fire master	11 (46)	5 (63)	1.93	0.29, 15.34	0.69
Location of sample					
Indoor	11 (46)	5 (63)	1.00	Reference	
Outdoor	13 (54)	3 (38)	0.52	0.07, 3.39	0.69
Size of house, 50 feet^2^			1.00	0.30, 3.33	1.00
Missing	3	1			
Size of house, m^2^			1.00	0.77, 1.30	1.00
Missing	3	1			
How long lived in house, two months			1.09	0.88, 1.32	0.43
Missing	3	1			
How many people live in house			1.02	0.67, 1.54	0.93
Missing	7	1			
Occupant density, 10 residents/100 m^2^			1.03	0.84, 1.22	0.78
Missing	8	2			
How many children 0–18 years-old live in house					
0	12 (57)	6 (86)	1.00	Reference	
1–3	9 (43)	1 (14)	0.23	<0.01, 2.49	0.37
Missing	3	1			
How many children under 6 years-old live in house					
0	12 (63)	6 (86)	1.00	Reference	
1–3	7 (37)	1 (14)	0.30	0.01, 3.35	0.55
Missing	5	1			
Primary fuel used for cooking					
Gas only	6 (26)	0 (0)	0.37 ^b^	0.00, 2.38	
Wood only	13 (57)	5 (71)	1.00	Reference	
Other ^c^	4 (17)	2 (29)	1.29	0.09, 12.96	0.49
Missing	1	1			
Type of heating source in the home					
Electricity	12 (63)	4 (80)	1.00	Reference	
Other ^d^ or none	7 (37)	1 (20)	0.44	0.01, 5.79	0.89
Missing	5	3			
Type of non-electric light source in the home					
Candle	11 (52)	3 (43)	0.56	0.04, 9.11	
Generator	6 (29)	2 (29)	0.69	0.03, 13.30	
Other ^e^ or none	4 (19)	2 (29)	1.00	Reference	1.00
Missing	3	1			
Any smokers living in the home					
No	8 (35)	2 (29)	1.00	Reference	
Yes	15 (65)	5 (71)	1.32	0.16, 16.87	1.00
Missing	1	1			
How many smokers living in the home					
0	8 (42)	2 (40)	1.00	Reference	
1–2	6 (32)	2 (40)	1.31	0.07, 23.23	
3–4	5 (26)	1 (20)	0.81	0.01, 19.75	1.00
Missing	5	3			
How many smokers living in the home regularly smoke inside the home					
0–1	10 (71)	2 (50)	1.00	Reference	
2–4	4 (29)	2 (50)	2.36	0.13, 44.12	0.81
Missing	10	4			

Abbreviations: CO_2_, carbon dioxide; CI, confidence interval; LDL, lower detection limit; OR, odds ratio. ^a^ Estimated using simple (i.e., unadjusted) exact unconditional logistic regression models. ^b^ Median unbiased estimate. ^c^ Includes coal and wood, gas and wood. ^d^ Includes lightbulb, line cable. ^e^ Includes candle and torch, fuel.

**Table 4:** Associations between characteristics and SiO_2_ (quartz) air concentrations measured inside homes at brick kilns in Bhaktapur, Kathmandu Valley, Nepal, May 2018.

	SiO_2_ (Quartz), μg/m^3^				
Characteristic	Below LDL, *n* (%)	Above LDL, *n* (%)	GM ^a^	95% CI ^a^	*p*-Value ^a^
Kiln number					
1	0 (0)	8 (42)	9.26	5.62, 15.27	
2	2 (15)	6 (32)	6.69	3.97, 11.27	
3	4 (31)	4 (21)	7.27	4.09, 12.90	
4	7 (54)	1 (5)	2.67	1.20, 5.93	0.08
Type of home					
Worker	8 (62)	8 (42)	4.98	3.23, 7.66	
Fire master	5 (38)	11 (58)	7.75	5.25, 11.43	0.13
Location of sample					
Indoor	6 (46)	10 (53)	6.00	3.94, 9.13	
Outdoor	7 (54)	9 (47)	6.48	4.19, 10.00	0.80
Size of house, 50 feet^2^			1.08 ^b^	0.64, 1.83 ^b^	0.78
Missing	0	4			
Size of house, m^2^			1.01 ^b^	0.91, 1.14 ^b^	0.80
Missing	0	4			
How long lived in house, two months			1.09 ^b^	1.02, 1.18 ^b^	0.01
Missing	1	3			
How many people live in house			1.10 ^b^	0.91, 1.32 ^b^	0.32
Missing	2	6			
Occupant density, 10 residents/100 m^2^			0.97 ^b^	0.87, 1.08 ^b^	0.55
Missing	2	8			
How many children 0–18 years-old live in house					
0	9 (75)	9 (56)	5.64	3.59, 8.86	
1–3	3 (25)	7 (44)	6.56	3.81, 11.31	0.67
Missing	1	3			
How many children under 6 years-old live in house					
0	9 (75)	9 (64)	5.63	3.57, 8.90	
1–3	3 (25)	5 (36)	5.32	2.84, 9.96	0.88
Missing	1	5			
Primary fuel used for cooking					
Gas only	4 (31)	2 (12)	3.88	1.73, 8.69	
Wood only	8 (62)	10 (59)	6.39	4.16, 9.80	
Other ^c^	1 (8)	5 (29)	7.25	3.72, 14.12	0.46
Missing	0	2			
Type of heating source in the home					
Electricity	12 (100)	4 (33)	3.77	2.48, 5.75	
Other ^d^ or none	0 (0)	8 (67)	10.15	6.84, 15.07	0.0008
Missing	1	7			
Type of non-electric light source in the home					
Candle	8 (67)	6 (38)	5.37	3.20, 8.99	
Generator	0 (0)	8 (50)	9.26	5.27, 16.28	
Other ^e^ or none	4 (33)	2 (13)	4.04	1.79, 9.09	0.20
Missing	1	3			
Any smokers living in the home					
No	2 (15)	8 (47)	9.36	5.78, 15.13	
Yes	11 (85)	9 (53)	4.75	3.16, 7.14	0.03
Missing	0	2			
How many smokers living in the home					
0	2 (22)	8 (53)	9.43	6.04, 14.75	
1–2	5 (56)	3 (20)	3.53	1.91, 6.54	
3–4	2 (22)	4 (27)	7.52	4.15, 13.61	0.03 ^f^
Missing	4	4			
How many smokers living in the home regularly smoke inside the home					
0–1	8 (80)	4 (50)	4.14	2.27, 7.55	
2–4	2 (20)	4 (50)	6.11	3.17, 11.79	0.37
Missing	3	11			

Abbreviations: CI, confidence interval; GM, geometric mean; LDL, lower detection limit; SiO_2_, crystalline silica. ^a^ Estimated using simple (i.e., unadjusted) Tobit regression models of the natural logarithm transformed values. ^b^ Exponentiated regression coefficient and 95% CI (i.e., GM SiO_2_ [quartz] concentration ratio for a specified change in the independent variable or exp(β)—1 = percent change in GM SiO_2_ [quartz] concentration for a specified change in the independent variable). ^c^ Includes coal and wood, gas and wood. ^d^ Includes lightbulb, line cable. ^e^ Includes candle and torch, fuel. ^f^ Adjusting for multiple comparisons using the Tukey–Kramer method, tests of pairwise differences among categories of how many smokers living in the home had the following *p*-values: 0 vs. 1–2: 0.01, 0 vs. 3–4: 0.55, and 1–2 vs. 3–4: 0.08.

## Data Availability

The data presented in this study are available upon request from the corresponding author.

## Supplementary Materials

The following supporting information can be downloaded at: https://www.mdpi.com/article/10.3390/ijerph191912431/s1, Table S1. Associations between characteristics and having CO_2_ air concentrations measured inside homes above the LDL at brick kilns in Bhaktapur, Kathmandu Valley, Nepal, May 2018; Table S2. Associations between characteristics and SiO_2_ (quartz) air concentrations measured inside homes at brick kilns in Bhaktapur, Kathmandu Valley, Nepal, May 2018.
