# Supplementary material for: Gaseous Air Pollutants and Respirable Crystalline Silica Inside and Outside Homes at Brick Kilns in Bhaktapur, Kathmandu Valley, Nepal
Source: Int J Environ Res Public Health. 2022 Sep 29;19(19):12431. doi: 10.3390/ijerph191912431 (PMC9566599; doi:10.3390/ijerph191912431)
Supplement: Supplementary file 1 [file ijerph-19-12431-s001.zip › ijerph-1871789-supplementary.pdf]

Supplementary Materials:

Gaseous Air Pollutants and Respirable Crystalline Silica Inside and Outside Homes at Brick Kilns in Bhaktapur, Kathmandu Valley, Nepal

John D. Beard [1,*], Steven M. Thygerson [1], Alisandra Olivares [1], Jaxson E. Tadje [1], Selah Willis [1] and James D. Johnston [1]

[1] Department of Public Health, Brigham Young University, Provo, UT 84602, USA
* Correspondence: john_beard@byu.edu; Tel.: +1-801-422-7491

Table S1. Associations between characteristics and having CO_2 air concentrations measured inside homes above the LDL at brick kilns in Bhaktapur, Kathmandu Valley, Nepal, May 2018.

Characteristic	CO_2, ppm				
	Below LDL, n (%)	Above LDL, n (%)	Exact OR[a]	Exact 95% CI[a]	Exact p-Value[a]
Kiln number					
1	3 (27)	1 (20)	1.00	Reference	
2	3 (27)	1 (20)	1.00	0.01, 104.37	
3	3 (27)	1 (20)	1.00	0.01, 104.37	
4	2 (18)	2 (40)	2.60	0.08, 235.00	1.00
Type of home					
Worker	6 (55)	2 (40)	1.00	Reference	
Fire master	5 (45)	3 (60)	1.73	0.14, 28.99	1.00
Size of house, 50 feet^2			1.13	0.23, 5.92	0.88
Missing	1	1			
Size of house, m^2			1.03	0.72, 1.47	0.88
Missing	1	1			
How long lived in house, two months			0.05	< 0.01, 1.00	0.05
Missing	1	1			
How many people live in house			1.17	0.66, 2.15	0.63
Missing	3	1			
Occupant density, 10 residents/100 m^2			1.13	0.86, 1.50	0.45
Missing	3	2			
How many children 0-18 years-old live in house					
0	6 (60)	3 (75)	1.00	Reference	
1-3	4 (40)	1 (25)	0.52	0.01, 9.80	1.00
Missing	1	1			
How many children under 6 years-old live in house					
0	6 (67)	3 (75)	1.00	Reference	

Characteristic	CO_2, ppm				
	Below LDL, n (%)	Above LDL, n (%)	Exact OR[a]	Exact 95% CI[a]	Exact *p*-Value[a]
1-3	3 (33)	1 (25)	0.69	0.01, 14.34	1.00
Missing	2	1			
Primary fuel used for cooking					
Gas only	3 (27)	0 (0)	0.42[b]	0.00, 3.35	
Wood only	5 (45)	4 (100)	1.00	Reference	
Other[c]	3 (27)	0 (0)	0.42[b]	0.00, 3.35	0.39
Missing	0	1			
Type of heating source in the home					
Electricity	5 (63)	3 (75)	1.00	Reference	
Other[d] or none	3 (38)	1 (25)	0.58	0.01, 12.43	1.00
Missing	3	1			
Type of non-electric light source in the home					
Candle	6 (60)	1 (25)	0.12	< 0.01, 4.05	
Generator	3 (30)	1 (25)	0.22	< 0.01, 8.85	
Other[e] or none	1 (10)	2 (50)	1.00	Reference	0.25
Missing	1	1			
Any smokers living in the home					
No	5 (45)	0 (0)	1.00	Reference	
Yes	6 (55)	4 (100)	3.51[b]	0.49, ∞	0.15
Missing	0	1			
How many smokers living in the home					
0	5 (55)	0 (0)	1.00	Reference	
1-2	2 (22)	2 (67)	3.77[b]	0.40, ∞	
3-4	2 (22)	1 (33)	1.67[b]	0.09, ∞	0.27
Missing	2	2			
How many smokers living in the home regularly smoke inside the home					
0-1	4 (80)	2 (50)	1.00	Reference	
2-4	1 (20)	2 (50)	3.39	0.11, 293.83	0.81
Missing	6	1			

Abbreviations: CO_2, carbon dioxide; CI, confidence interval; LDL, lower detection limit; OR, odds ratio. [a] Estimated using simple (i.e., unadjusted) exact unconditional logistic regression models. [b] Median unbiased estimate. [c] Includes coal and wood, gas and wood. [d] Includes lightbulb, line cable. [e] Includes candle and torch, fuel.

Table S2. Associations between characteristics and SiO_2 (quartz) air concentrations measured inside homes at brick kilns in Bhaktapur, Kathmandu Valley, Nepal, May 2018.

Characteristic	SiO_2 (quartz), μg/m³				
	Below LDL, n (%)	Above LDL, n (%)	GM[a]	95% CI[a]	*p*-Value[a]
Kiln number					
1	0 (0)	4 (40)	7.77	4.25, 14.21	
2	1 (17)	3 (30)	7.70	4.12, 14.39	
3	2 (33)	2 (20)	5.65	2.89, 11.07	
4	3 (50)	1 (10)	4.27	1.95, 9.38	0.60
Type of home					
Worker	3 (50)	5 (50)	5.62	3.53, 8.94	
Fire master	3 (50)	5 (50)	7.17	4.53, 11.35	0.45
Size of house, 50 feet²			1.03[b]	0.57, 1.86[b]	0.92
Missing	0	2			
Size of house, m²			1.01[b]	0.89, 1.14[b]	0.93
Missing	0	2			
How long lived in house, two months			1.08[b]	0.99, 1.17[b]	0.09
Missing	0	2			
How many people live in house			1.14[b]	0.90, 1.44[b]	0.28
Missing	0	4			
Occupant density, 10 residents/100 m²			1.00[b]	0.87, 1.14[b]	0.95
Missing	0	5			
How many children 0-18 years-old live in house					
0	5 (83)	4 (50)	5.72	3.33, 9.80	
1-3	1 (17)	4 (50)	6.11	3.26, 11.44	0.87
Missing	0	2			
How many children under 6 years-old live in house					
0	5 (83)	4 (57)	5.58	3.12, 9.98	
1-3	1 (17)	3 (43)	5.44	2.55, 11.61	0.96
Missing	0	3			
Primary fuel used for cooking					
Gas only	1 (17)	2 (22)	6.98	3.13, 15.57	
Wood only	4 (67)	5 (56)	6.06	3.70, 9.92	
Other[c]	1 (17)	2 (22)	6.15	2.74, 13.78	0.96
Missing	0	1			
Type of heating source in the home					
Electricity	5 (100)	3 (43)	4.64	2.86, 7.53	
Other[d] or none	0 (0)	4 (57)	9.74	5.70, 16.66	0.04
Missing	1	3			
Type of non-electric light source in the home					
Candle	3 (60)	4 (44)	6.19	3.66, 10.48	
Generator	0 (0)	4 (44)	7.77	4.13, 14.62	

Characteristic	SiO$_2$ (quartz), μg/m^3				
	Below LDL, n (%)	Above LDL, n (%)	GM[a]	95% CI[a]	*p*-Value[a]
Other[e] or none	2 (40)	1 (11)	6.21	2.65, 14.57	0.85
Missing	1	1			
Any smokers living in the home					
No	1 (17)	4 (44)	8.14	4.53, 14.60	
Yes	5 (83)	5 (56)	5.46	3.41, 8.73	0.29
Missing	0	1			
How many smokers living in the home					
0	1 (25)	4 (50)	8.32	5.30, 13.07	
1-2	2 (50)	2 (25)	4.40	2.47, 7.83	
3-4	1 (25)	2 (25)	9.06	5.01, 16.39	0.14
Missing	2	2			
How many smokers living in the home regularly smoke inside the home					
0-1	3 (75)	3 (60)	5.93	3.00, 11.71	
2-4	1 (25)	2 (40)	5.25	2.14, 12.83	0.83
Missing	2	5			

Abbreviations: CI, confidence interval; GM, geometric mean; LDL, lower detection limit; SiO$_2$, crystalline silica. [a] Estimated using simple (i.e., unadjusted) Tobit regression models of the natural logarithm transformed values. [b] Exponentiated regression coefficient and 95% CI (i.e., GM SiO$_2$ [quartz] concentration ratio for a specified change in the independent variable or $\exp(\beta) - 1$ = percent change in GM SiO$_2$ [quartz] concentration for a specified change in the independent variable). [c] Includes coal and wood, gas and wood. [d] Includes lightbulb, line cable. [e] Includes candle and torch, fuel.
